# Selective and Preferential Separation of Rhodium (III) from Palladium (II) and Platinum (IV) Using a *m*-Phenylene Diamine-Containing Precipitant

**DOI:** 10.1038/s41598-019-48920-9

**Published:** 2019-08-27

**Authors:** Kazuya Matsumoto, Sumito Yamakawa, Kazutoshi Haga, Katsuyuki Ishibashi, Mitsutoshi Jikei, Atsushi Shibayama

**Affiliations:** 1 Department of Materials Science, Graduate School of Engineering Science, Akita University, 1-1 Tegatagakuen-machi, Akita-shi, Akita 010-8502 Japan; 2 Department of Earth Resource Engineering and Environmental Science, Graduate School of International Resource Science, Akita University, 1-1 Tegatagakuen-machi, Akita-shi, Akita 010-8502 Japan

**Keywords:** Environmental sciences, Environmental chemistry

## Abstract

Although Rh is an industrially important and the most expensive platinum group metal (PGM), the selective and preferential separation of Rh from PGM mixtures still remains as a big challenge. In this work, the separation of Rh (III) from Pd (II) and Pt (IV) in a hydrochloric acid (HCl) solution was studied using a *m*-phenylene diamine-containing precipitant (*m*-**PDA**). At high HCl concentrations (6.0–8.0 M), most of the Rh (III) (>90%) was precipitated, and Pd (II) and Pt (IV) were hardly precipitated (<5%). On the other hand, over 85% of Pd (II) and Pt (IV) precipitated along with small amount of Rh (III) (<25%) at low HCl concentrations (1.0–2.0 M). As a consequence, *m*-**PDA** enabled selective and preferential precipitation of Rh (III) at high HCl concentrations. XPS and TG analyses revealed that the Rh-containing precipitate is an ion-pair complex composed of one [RhCl_6_]^3−^ anion and three *m*-**PDA** cations. The Rh desorption from the precipitate as well as the recovery of *m*-**PDA** was successfully achieved using an NH_4_OH solution. This method is a promising practical approach to Rh recovery.

## Introduction

Rhodium (Rh) is a member of the platinum group metals (PGMs), and this element is industrially important because it displays the highest electrical and thermal conductivities among PGMs; it is also very thermally stable, and in ionic form, it is characterised by high catalytic activity^1–4^. To date, Rh has been mainly used in automotive catalytic converters along with palladium (Pd) and platinum (Pt)^5–7^. Since Rh is currently the most expensive PGM, selective and preferential recovery of this metal from PGM mixtures is a quite important goal from the viewpoint of economic efficiency.

Generally, the separation and recovery of PGM ions are performed by solvent extraction from metal-ion-containing aqueous solutions^8–12^. For example, extraction of Pd (II) and Pt (IV) from hydrochloric acid (HCl) solutions can be achieved using di-*n*-octyl sulfide^13^ and tri-*n*-butyl phosphate^14^ as extracting agents, respectively. However, no practical extracting agent exists to date for Rh (III), and in most cases, Rh is recovered from the raffinate after the extraction procedures for the recovery of the other PGMs have been implemented. This approach is adopted even in major industrial plants, like the Vale Acton precious metal refinery^15^. However, leaving Rh recovery as the final step of the PGM recovery process is an economically inefficient strategy. Furthermore, Rh recovery from the raffinate is an undesirable approach from the standpoint of the grade of the recovered metal because the raffinate contains small amounts of other metals that cannot be removed during the recovery process.

The recovery of PGMs from HCl solutions has been widely studied because PGMs in spent catalysts can be leached in HCl medium^16,17^. It is known that PGMs are generally recovered via a ligand–metal coordination mechanism or an ion-pair formation mechanism^18^. In the ligand–metal coordination mechanism, the extraction of metals generally occurs on the order of Pd (II)≫ Rh (III)≫ Pt (IV); in fact, Rh (III) and Pt (IV) are considered to be kinetically inert^19^. Furthermore, the order of extractability of PGM ions via ion-pair formation for chloro-complexes formed in aqueous HCl solution is reported to be [MCl_4_]^2−^ ≅ [MCl_6_]^2−^ > [MCl_6_]^3−^ > aqua species, like [MCl_4_(H_2_O)_2_]^−^ ^20^. Although [PdCl_4_]^2−^ and [PtCl_6_]^2−^ can be extracted by an approach based on ion-pair formation, [RhCl_6_]^3−^ and the aqua chloro-complexes of Rh (III), like [RhCl_4_(H_2_O)_2_]^−^ and [RhCl_5_(H_2_O)]^2−^, are quite difficult to extract. Therefore, selective and preferential recovery of Rh is widely recognised as a supremely difficult goal to achieve^21^.

Narita *et al*. reported that specifically designed compounds, tertiary amines containing two or three *N*-disubstituted amide groups, act as Rh (III) extracting agents, and these nitrogen compounds were successfully used for the separation of Rh (III) from Pd (II) and Pt (IV)^22,23^. However, the separation of Rh (III) required two steps: (1) extraction of Pd (II), Pt (IV) and Rh (III) from an HCl solution into an organic phase and (2) back-extraction of Rh (III) into a concentrated HCl solution. This separation method exploits the lower extractability level of Rh (III) (i.e. the higher back-extractability of this ion) with respect to Pd (II) and Pt (IV); additionally, this approach does not allow achieving the preferential extraction of Rh (III) in a single step. It has been reported that the addition of SnCl_2_ has positive effect on the extraction of Rh (III)^24–26^. Although tertiary amines and organophosphines act as extractants for Rh (III) from SnCl_2_-containing HCl solutions, co-extraction of Pt (IV) cannot be suppressed. Rh (III) separation has also been performed using ion exchange resins^27–30^. Since Rh (III) in HCl solutions is nearly inert for ion-pair formation as mentioned above, only Rh (III) can be eluted from the anion exchange resins despite the adsorption of Pd (II) and Pt (IV) on the resins. Rh recovery methods up to this point are based on the inert nature of Rh (III) towards coordination and ion pair formation.

Recently, we have developed a method that enables the selective precipitation of Pd (II) or Pt (IV) from HCl solutions based on the use of primary aromatic amines as precipitants^31,32^. In comparison with conventional solvent extractions, this method has the advantage that no organic solvents are needed for it. We have reported that the *m*-phenylene diamine-containing precipitant (***m*-PDA**) can form ion-pairs with [PdCl_4_]^2−^ and [PtCl_6_]^2−^ in 0.1 M HCl^32^. Since Rh (III) forms chloro-complex anions in HCl, we expected that ***m*-PDA** also forms ion-pairs with chloro-complex anions of Rh (III) to recover Rh (III) as a precipitate. Herein, we present a procedure for the selective and preferential precipitation of Rh (III) from a mixture of Pd (II), Pt (IV) and Rh (III) in HCl, which relies on the use of ***m*-PDA**.

## Results and Discussion

**Figure 1:**
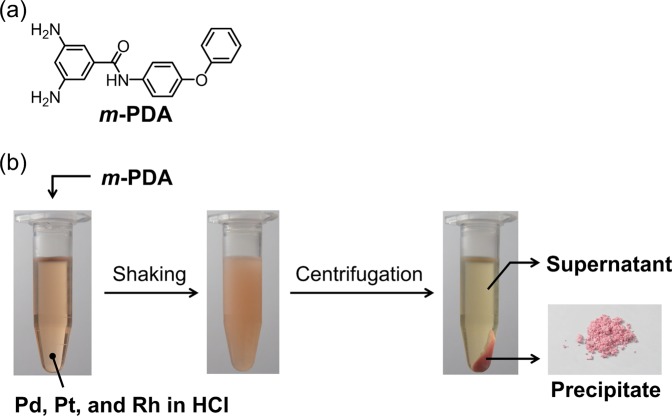
(**a**) Chemical structure of ***m*-PDA**. (**b**) Schematic representation of the procedure for Rh (III) precipitation from HCl solutions containing Pd (II), Pt (IV) and Rh (III). The photographs are from an experiment run in the following conditions: 8 M HCl, ***m*-PDA**/Rh = 15 mol/mol and 3 h of shaking.

**Figure 2:**
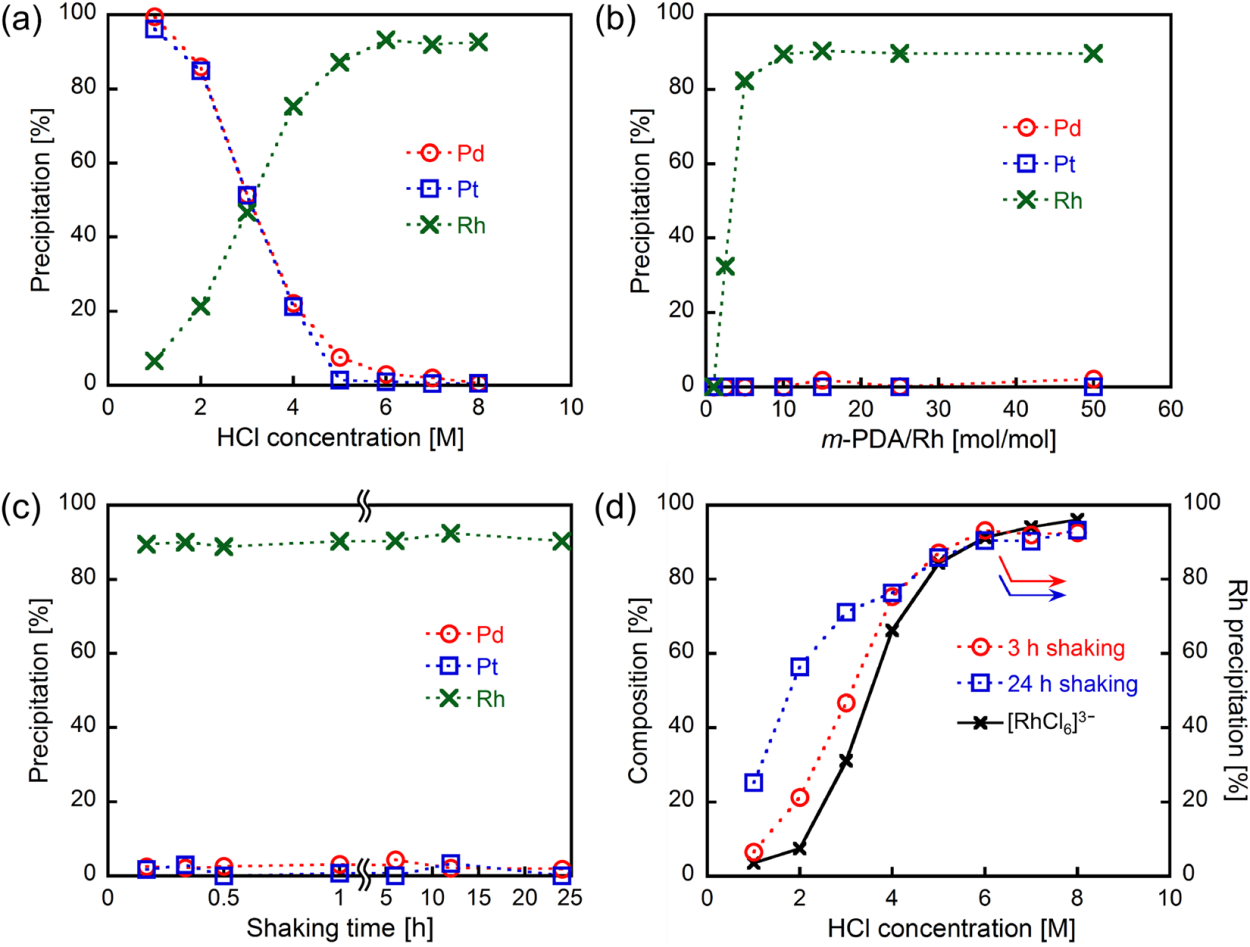
(**a**) Effect of HCl concentrations on the metal precipitation after 3 h of shaking. (**b**) Effect of the ***m*-PDA** loading on the metal precipitation. HCl concentration = 8 M, shaking time = 3 h. (**c**) Effect of the shaking time on the metal precipitation. HCl concentration = 8 M, ***m*-PDA**/Rh = 15 mol/mol. (**c**) Effect of HCl concentrations on the distribution of Rh (III) chloro-complex anions and Rh (III) precipitation (***m*-PDA**/Rh = 15 mol/mol).

In the present study, we have selected ***m*-PDA** (Fig. 1a) as a precipitant instead of *m*-phenylene diamine. This is because ***m*-PDA** is suitably balanced in hydrophilicity and hydrophobicity in HCl, while hydrophilicity of *m*-phenylene diamine in HCl is too high to be suitable for metal precipitation. ***m*-PDA** was synthesized according to our previous report^32^, and the differential precipitation of metals was carried out by implementing the following procedure (Fig. 1b): to the HCl solution containing Pd (II), Pt (IV) and Rh (III) (1.0 mmol/L each), ***m*-PDA** was added. The resulting mixture was shaken vigorously. A precipitate formed as a consequence, which was separated from the supernatant by centrifugation. The precipitation percentages of the metals were evaluated by inductively coupled plasma atomic emission spectroscopy (ICP-AES) experiments conducted on the supernatant. Figure 2a shows the precipitation behaviours of Pd (II), Pt (IV) and Rh (III) as a function of HCl concentration. After the described procedure was conducted in a 1.0–2.0 M HCl solution, over 85% of the Pd and Pt ions precipitated, whereas less than 25% of Rh did. This behaviour at low HCl concentrations corresponds to what we previously reported: Pd (II) and Pt (IV) had quantitatively (>95%) precipitated using ***m*-PDA** in a 0.1 M HCl solution^32^. Notably, the precipitation percentages of Pd and Pt ions decreased as the concentration of HCl increased, whereas that of Rh (III) increased in a complementary fashion. At high HCl concentrations (6.0–8.0 M), over 90% of Rh (III) precipitated, whereas both Pd (II) and Pt (IV) hardly did (<5% precipitation). As the photographs in Fig. 1b show, the resulting precipitate has a pinkish colour, which corresponds to the colour of a Rh (III) HCl solution. These results clearly indicate that preferential, selective and efficient precipitation of Rh (III) can be achieved at high HCl concentrations using ***m*-PDA** as precipitant.

Metal precipitation experiments using 8 M HCl solutions were carried out by changing the loading amount of ***m*-PDA** to find out the optimal feed ratio (Fig. 2b). The percentage of Rh (III) ions precipitated increased as ***m*-PDA** loading increased, and it reached a plateau at ***m*-PDA**/Rh (III) = 10 mol/mol. On the other hand, neither the Pd nor the Pt ion precipitated even at the high feed ratio of ***m*-PDA**/Rh (III) = 50 mol/mol. The effect of the mixture shaking time on metal precipitation was investigated under conditions whereby ***m*-PDA**/Rh (III) = 15 mol/mol in 8 M HCl (Fig. 2c). Rh (III) precipitation was completed after just a few minutes of shaking. Furthermore, the composition of the precipitated metal ions stayed unchanged and was independent of the shaking time.

It is known that Rh (III) exists in HCl in the form of chloro-complex anions, such as [RhCl_4_(H_2_O)_2_]^−^, [RhCl_5_(H_2_O)]^2−^ and [RhCl_6_]^3−^, and these species’ composition changes with the concentration of HCl^21,33^. Figs S1 and S2 show the UV–Vis absorption spectra of a Rh (III) solution at different HCl concentrations and the distribution of Rh (III)-based chloro-complex anionic species determined by UV–Vis spectroscopy, respectively^33,34^. The predominant species at low HCl concentrations is [RhCl_5_(H_2_O)]^2−^, whereas the proportion of [RhCl_6_]^3−^ increases along with that of HCl, and this species becomes the prevailing one at high HCl concentrations (>4 M). Figure 2d shows the precipitation behaviour of Rh (III) with ***m*-PDA** as precipitant, as well as the distribution of Rh (III)-based chloro-complex anions. In comparison with the distribution of Rh (III)-based species, the Rh precipitation percentages after 3 h of shaking are matched with the composition of [RhCl_6_]^3−^. This fact indicates that ***m*-PDA** selectively prompts the precipitation of [RhCl_6_]^3−^, regardless of HCl concentration. At HCl concentration in the 1.0–4.0 M range, the percentage of Rh ions precipitated after 24 h of shaking is clearly higher than that measured after 3 h of shaking. This observation would be the result of the equilibrium shift from [RhCl_4_(H_2_O)_2_]^−^ and [RhCl_5_(H_2_O)]^2−^ to [RhCl_6_]^3−^.

**Figure 3:**
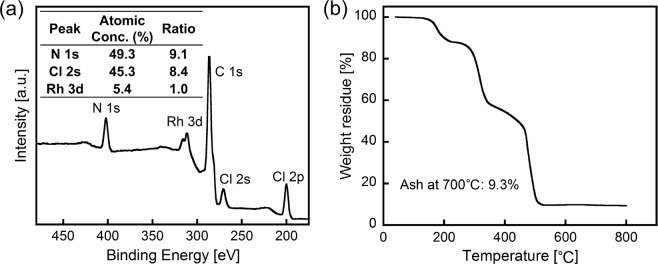
(**a**) XPS spectrum of the Rh-containing precipitate obtained from 8 M HCl solutions. The inset shows the atomic ratios calculated from the XPS peaks. (**b**) TG curve of the Rh-containing precipitate at a heating rate of 10 °C/min under an air flow of 200 mL/min.

**Figure 4:**
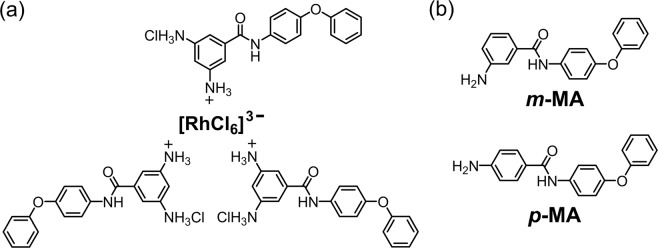
(**a**) Plausible ion-pair structure composed of [RhCl_6_]^3−^ and ***m*-PDA**. (**b**) Chemical structure of monoamine compounds.

To investigate the structure of the Rh (III)-containing precipitate, X-ray photoelectron spectroscopy (XPS) and thermogravimetric (TG) measurements were carried out. The Rh (III)-containing precipitate obtained by adding ***m*-PDA** to an 8 M HCl solution of Rh (III) was used for these measurements. As the data reported in Fig. 3a show, characteristic XPS peaks of Rh 3d, N 1s, Cl 2s and Cl 2p were clearly observed, indicating that the precipitate is a complex comprising Rh and ***m*-PDA**. The N/Rh atomic ratio calculated from the XPS peaks was 9.1. This result indicates that the Rh-containing precipitate is composed of the [RhCl_6_]^3−^ ion and the ***m*-PDA** species, in a 1:3 stoichiometry, for a chemical unit containing a total of nine nitrogen atoms. Since the amine groups of ***m*-PDA** form ammonium cations in HCl, the Rh-containing precipitate is considered to be an ion-pair between [RhCl_6_]^3−^ and ***m*-PDA** cations. On the basis of the 1:3 stoichiometry between [RhCl_6_]^3−^ and ***m*-PDA**, the ion-pair would consist of one [RhCl_6_]^3−^ and three ***m*-PDA** monovalent cations, which would satisfy the condition of charge balance (Fig. 4a). The atomic ratio of the plausible ion-pair structure (N:Cl:Rh = 9:9:1) is consistent with that calculated from the XPS peaks (N:Cl:Rh = 9.1:8.4:1.0). The TG curve of the Rh-containing precipitate under atmospheric conditions is reported in Fig. 3b. A weight loss resulting from the decomposition of ***m*-PDA** and the elimination of chlorine from the Rh-based species was observed below 600 °C, and the weight of the residue at 700 °C corresponded to 9.3% of the initial weight of the precipitate. According to literature data, below 900 °C, the combustion of RhCl_3_ in an oxidative atmosphere yields Rh_2_O_3_^35^. The expected weight fraction of Rh_2_O_3_ after the combustion of the plausible ion-pair reported in Fig. 4a is 9.2%, which is remarkably close to the measured weight of the 700 °C residue as a percentage of the Rh-containing precipitate. This result strongly supports the presence of the hypothesised ion-pair structure: a complex made up of one [RhCl_6_]^3−^ anion and three ***m*-PDA** (monovalent) cations.

We also analyzed the precipitate obtained from a Rh-containing 2 M HCl solution—in which a major Rh (III)-containing species is [RhCl_5_(H_2_O)]^2−^—by XPS and TG measurements. The inferred atomic composition (Rh, N and Cl) and the weight of the residue at 700 °C were almost the same as those measured for the precipitate obtained from the 8 M HCl solution (Fig. S3). This fact clearly indicates that ***m*-PDA** can selectively prompt the precipitation of [RhCl_6_]^3−^ via formation of ion-pairs composed of one [RhCl_6_]^3−^ and three ***m*-PDA** cations.

**Figure 5:**
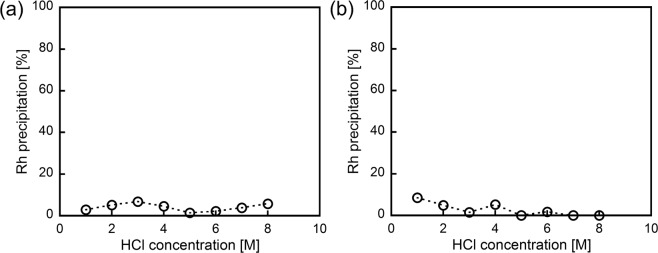
Effect of HCl concentrations on the Rh precipitation using (**a**) ***m*-MA** and (**b**) ***p*-MA**. ***m*-MA**/Rh or ***p*-MA**/Rh = 30 mol/mol, 3 h of shaking.

As can be evinced from the structure depicted in Fig. 4a, one of the two ammonium groups in ***m*-PDA** is used for the formation of the ion-pair with [RhCl_6_]^3−^. To determine whether the diamine structure of ***m*-PDA** is necessary to obtain the Rh-based precipitate, Rh precipitation experiments were carried out using the monoamine compounds ***m*-MA** and ***p*-MA**, reported in Fig. 4b, as analogues of ***m*-PDA**. Using these compounds, Rh (III) precipitation did not occur (only <10% of Rh (III) precipitated), regardless of HCl concentration (Fig. 5). Thus, the diamine structure of ***m*-PDA** is a requirement for Rh (III) precipitation. Presumably, the two main reasons for the successful precipitation of Rh (III) prompted by ***m*-PDA** are the following: (1) the primary ammonium cations in ***m*-PDA** have relatively small steric hindrance, and (2) the two ammonium cations in ***m*-PDA** display a high enough hydrophilicity to form the ion-pair by overcoming the large hydration shell of [RhCl_6_]^3−^. Generally, the steric hindrance of conventional extracting agents, such as secondary, tertiary and quaternary ammonium cations, is assumed to prevent the formation of ion-pairs with a trivalent anion like [RhCl_6_]^3−^ ^36^; therefore, a primary ammonium cation with relatively small steric hindrance would be favoured in ion-pair formation. Furthermore, it is known that [RhCl_6_]^3−^ has a larger hydration shell than [PdCl_4_]^2−^ and [PtCl_6_]^2−^ ^37^; therefore, the hydrophilicity of monoammonium compounds would not provide enough drive to overcome the hydration shell of [RhCl_6_]^3−^. As a result, only ***m*-PDA**, with its two ammonium cations, is able to form an ion-pair with [RhCl_6_]^3−^. It is noteworthy that chloro-complex anions of Rh (III) have been widely recognized as inert species for the recovery via ion-pair formation^18^. Therefore, Rh separation method in this work is totally different from conventional methods, and has great impact on the PGM recycling. In contradiction to the almost quantitative precipitation of Rh (III) at high HCl concentrations, Pd (II) and Pt (IV) were hardly precipitated using ***m*-PDA**. This would be caused by low stability of ion-pair complexes of Pd (II) and Pt (IV) with ***m*-PDA** at high HCl concentrations: ***m*-PDA** cations in these complexes are easily exchanged with protons from HCl. The detailed analysis on the stability of the ion-pair complexes is under studying.

**Figure 6:**
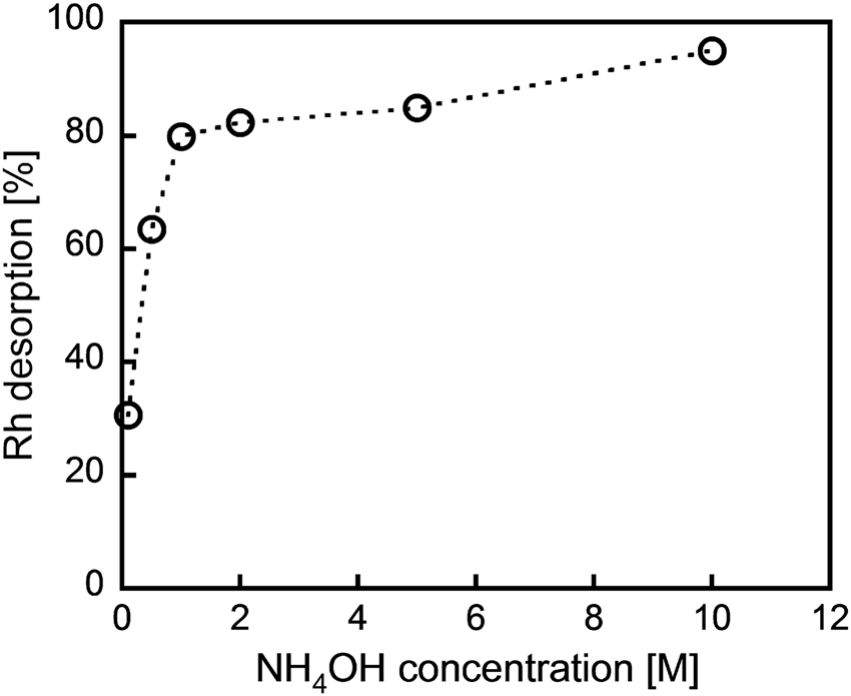
Effect of NH_4_OH concentration on the Rh desorption. Rh-containing precipitate/NH_4_OH = 1.4 mg/mL and 3 h of shaking.

To use our ***m*-PDA**-based Rh (III) precipitation method as a Rh recovery strategy, Rh desorption from the precipitate is necessary. We investigated the Rh desorption process by adding the Rh-containing precipitate into NH_4_OH solutions. The resulting solutions contained about 80%, in the case of 1 M NH_4_OH, and 95%, in the case of 10 M NH_4_OH, of the Rh originally present in the precipitate (Fig. 6). This successful desorption results from the collapse of the ion-pairs caused by the NH_4_OH-driven transformation of ***m*-PDA**’s ammonium cations into amine groups. It is worth noting that ***m*-PDA** was recovered as a solid residue after Rh desorption, and its chemical structure was unchanged (Fig. S4). Efficient Rh desorption and the reusability of ***m*-PDA** are currently still being investigated by our research group.

## Methods

### Materials

*N*,*N*-Dimethylacetamide (DMAc) was purchased from Wako Pure Chemical Industries Ltd. and distilled from calcium hydride before use. Thionyl chloride and NH_4_OH was purchased from Kanto Chemical Co., Inc., and used as received. 4-Phenoxyaniline was purchased from Tokyo Kasei Kogyo Co., Ltd. and used as received. ***m*-PDA**, 3-(Trifluoroacetamido)benzoic acid, and 4-(trifluoroacetamido)benzoic acid were prepared following methods reported in the literature^32^. Pd (II) and Pt (IV) standard solutions (1000 ppm in 1 M HCl) were purchased from Wako Pure Chemical Industries, Ltd. Rh (III) standard solution (1000 ppm in 1 M HCl) was purchased from Kanto Chemical Co., Inc.

### Synthesis of *m*-MA

3-(Trifluoroacetamido)benzoic acid (0.63 g, 2.71 mmol) in thionyl chloride (10 mL) was refluxed for 3 h. Thionyl chloride was removed by distillation and then chloroform was added. After distillation of chloroform, the resulting product was dried in vacuo and dissolved in DMAc (5 mL). 4-Phenoxyaniline (0.45 g, 2.46 mmol) was added to the former DMAc solution and then the mixture was stirred for 12 h at room temperature, followed by for 1 h at 50 °C under nitrogen. To the solution was added water (0.35 mL) and the mixture was stirred for another 1 h at 50 °C. After the addition of hydrazine monohydrate (1.4 mL), the mixture was stirred for 4 h at 50 °C. Then the mixture was poured into a 5% NaHCO_3_ aqueous solution, and the resulting precipitate was collected by filtration, washed with water, and subsequently dried for 12 h at 80 °C in vacuo. The yield was 0.72 g (96%). ^1^H NMR (DMSO-*d*_6_, δ, ppm): 5.30 (s, 2H), 6.75 (d, *J* = 7.8 Hz, 1H), 6.98 (d, *J* = 7.5 Hz, 2H), 7.02 (d, *J* = 9.5 Hz, 2H), 7.05–7.17 (m, 4H), 7.37 (t, *J* = 7.5 Hz, 2H), 7.78 (d, *J* = 9.0 Hz, 2H), 10.08 (s, 1H). ^13^C NMR (DMSO-*d*_6_, δ, ppm): 112.94, 114.66, 116.72, 117.81, 119.25, 121.85, 122.89, 128.71, 129.90, 135.27, 135.88, 148.72, 151.81, 157.38, 166.19.

### Synthesis of *p*-MA

***p*-MA** was synthesized in the same manner as ***m*-MA** although 4-(trifluoroacetamido)benzoic acid was used instead of 3-(trifluoroacetamido)benzoic acid. The yield was 96%. ^1^H NMR (DMSO-*d*_6_, δ, ppm): 5.73 (s, 2H), 6.60 (d, *J* = 8.5 Hz, 2H), 6.98 (d, *J* = 8.0 Hz, 2H), 7.00 (d, *J* = 9.0 Hz, 2H), 7.10 (t, *J* = 7.5 Hz, 1H), 7.37 (t, *J* = 7.5 Hz, 2H), 7.72 (d, *J* = 8.5 Hz, 2H), 7.76 (d, *J* = 9.0 Hz, 2H), 9.78 (s, 1H). ^13^C NMR (DMSO-*d*_6_, δ, ppm): 112.53, 117.74, 119.22, 121.04, 121.73, 122.82, 129.26, 129.89, 135.70, 151.42, 152.06, 157.47, 165.13.

### Metal precipitation experiments using *m*-PDA

To HCl solutions (1 mL) containing Pd (II), Pt (IV), and Rh (III) (1.0 mmol/L each) were added ***m*-PDA**, and the resulting mixtures were shaken vigorously at room temperature. After centrifugation (7200 *g*, 10 min), the metal concentrations in the supernatant were evaluated by ICP-AES. The HCl concentrations, the amount of precipitating agents, and the shaking time were changed in the metal precipitation experiments.

### Rh precipitation experiments using *m*-MA and *p*-MA

To HCl solutions (1 mL) containing Rh (III) (1.0 mmol/L) were added ***m*-MA** or ***p*-MA** (9.1 mg, 0.03 mmol) and the mixtures were shaken vigorously at room temperature. After centrifugation (7200 *g*, 10 min), the Rh concentration in the supernatant was evaluated by ICP-AES. The HCl concentrations were changed in the Rh precipitation experiments.

### Isolation of Rh-containing precipitate

To a HCl solution (2 M or 8 M, 10 mL) containing Rh (III) (2.0 mmol/L) were added ***m*-PDA** (64 mg, 0.2 mmol) and the mixture was shaken vigorously at room temperature. The resulting solid was collected by filtration and washed with a 2 M HCl solution. The solid was dried for 48 h at room temperature in vacuo.

### Rh desorption experiments

To the Rh-containing precipitate (1.4 mg) was added a NH_4_OH solution (1 mL) and the mixture was shaken vigorously for 3 h at room temperature. After centrifugation (7200 *g*, 10 min), the Rh concentration in the supernatant was evaluated by ICP-AES. The concentrations of NH_4_OH were changed in the Rh desorption experiments.

### Measurements

^1^H and ^13^C NMR spectra were recorded using a JEOL JNM-ECX 500 NMR spectrometer (Jeol Co., Tokyo, Japan). The metal concentrations were measured on an ICP-AES instrument (SPS5510, SII Nanotechnology Inc.). X-ray photoelectron spectroscopy (XPS) measurements were conducted on an AXIS-ULTRA X-ray photoelectron spectrometer (Kratos Analytical Ltd.). UV-Vis absorption spectra were collected using a UV-Vis spectrophotometer (Model V-550, Jasco co., Tokyo, Japan). Thermogravimetric analysis (TGA) measurements were carried out using a STA7300 (Hitachi High-Tech Science Co., Tokyo, Japan) at a heating rate of 10 °C min^−1^ under air flow (200 mL/min).

## Conclusions

In conclusion, we have developed a selective and preferential method for Rh recovery that makes use of ***m*-PDA** as precipitant. At high HCl concentrations (6.0–8.0 M), Rh (III) was selectively recovered as a pinkish solid from the mixture of Pd (II) and Pt (IV). The resulting Rh-containing precipitate is an ion-pair complex composed of one [RhCl_6_]^3−^ anion and three ***m*-PDA** cations. The Rh desorption from the precipitate as well as the recovery of ***m*-PDA** was achieved using an NH_4_OH solution. This method is a promising practical approach to Rh recovery.

## Supplementary information

Supplementary Information

## Data Availability

The datasets generated during and/or analysed during the current study are available from the corresponding author on reasonable request.
