# Supplementary material for: Selective and Preferential Separation of Rhodium (III) from Palladium (II) and Platinum (IV) Using a m-Phenylene Diamine-Containing Precipitant
Source: Sci Rep. 2019 Aug 27;9:12414. doi: 10.1038/s41598-019-48920-9 (PMC6712025; doi:10.1038/s41598-019-48920-9)
Supplement: Supplementary file 1 — Supplementary Information [file 41598_2019_48920_MOESM1_ESM.docx]

**Supplementary Information**

**Selective and Preferential Separation of Rhodium (III) from Palladium (II) and Platinum (IV) Using a *m*-Phenylene Diamine-Containing Precipitant**

Kazuya Matsumoto^1,^*, Sumito Yamakawa^1^, Kazutoshi Haga^2^, Katsuyuki Ishibashi^1^, Mitsutoshi Jikei^1^, Atsushi Shibayama^2^

^1^ Department of Materials Science, Graduate School of Engineering Science, Akita University, 1-1 Tegatagakuen-machi, Akita-shi, Akita 010-8502, Japan

^2^ Department of Earth Resource Engineering and Environmental Science, Graduate School of International Resource Science, Akita University, 1-1 Tegatagakuen-machi, Akita-shi, Akita 010-8502, Japan

* kmatsu@gipc.akita-u.ac.jp


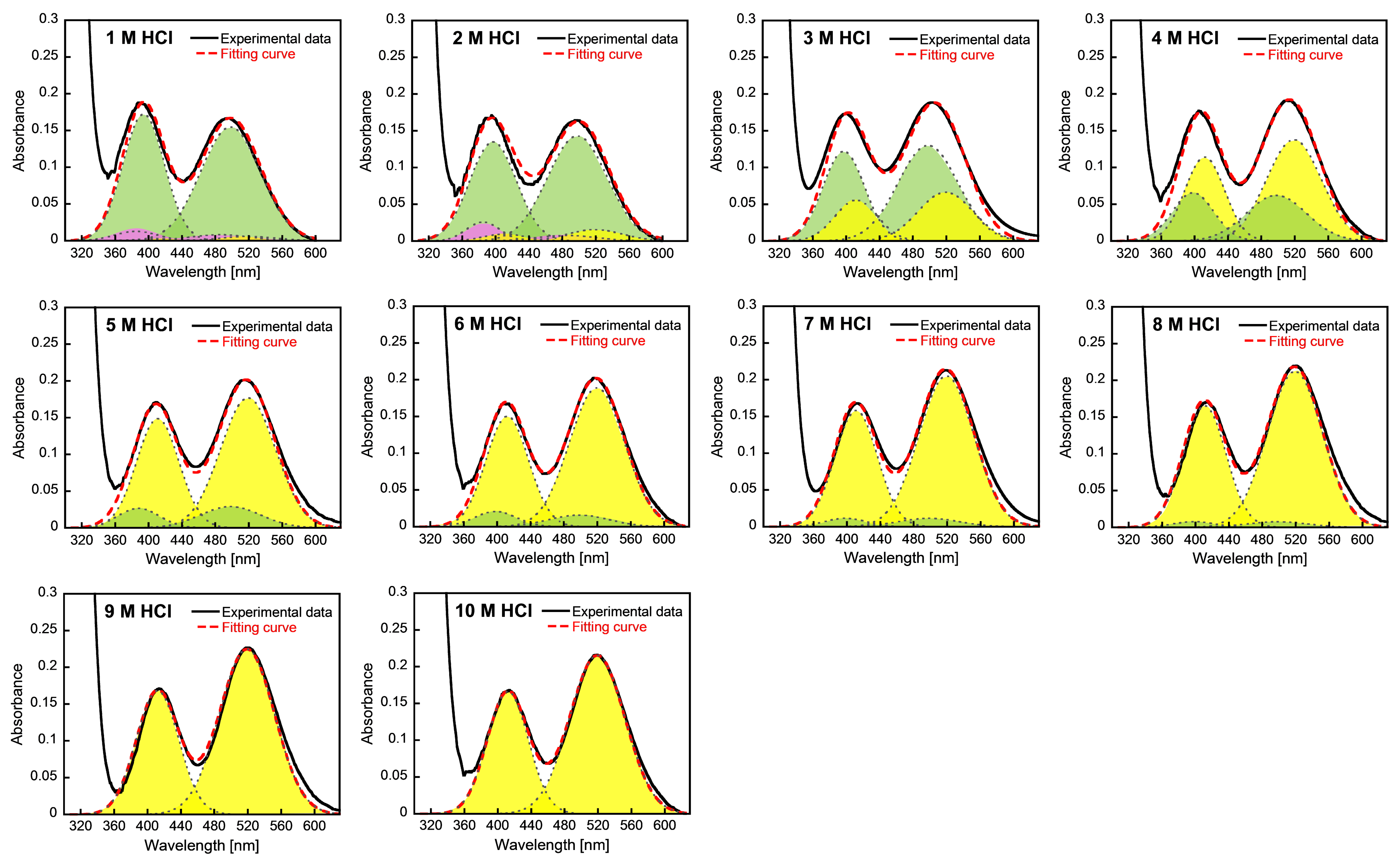


**Figure S1.** UV-Vis absorption spectra of Rh-containing HCl solution (2.0 mmol/L). [RhCl_4_(H_2_O)_2_]^−^, [RhCl_5_(H_2_O)]^2−^, and [RhCl_6_]^3−^ are shown in pink, green, and yellow, respectively.


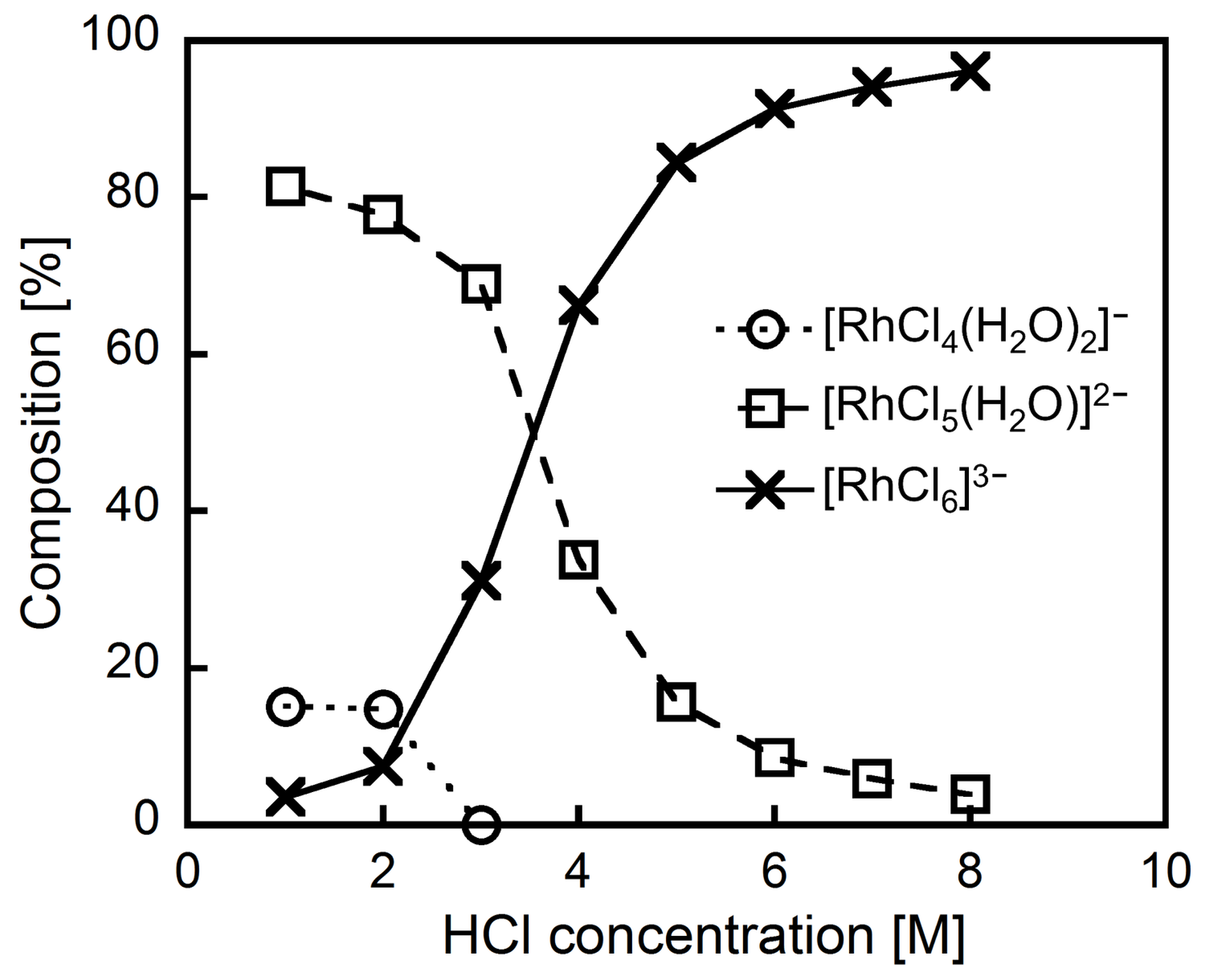


**Figure S2.** Distribution of Rh chloro-complex anions at different HCl concentrations.


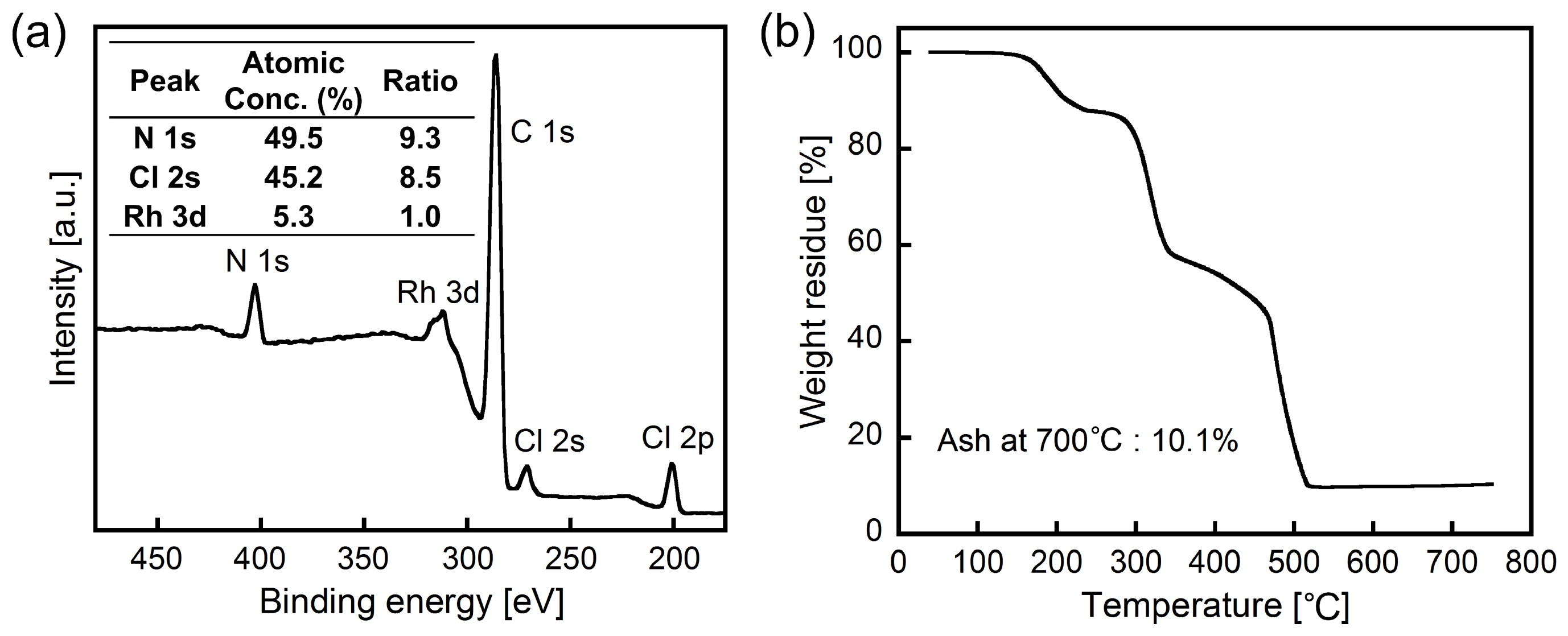


**Figure S3.** (a) XPS spectrum of the Rh-containing precipitate obtained from 2 M HCl solutions. The inset shows the atomic ratios calculated from the XPS peaks. (b) TG curve of the Rh-containing precipitate obtained from 2 M HCl solutions at a heating rate of 10 °C/min under an air flow of 200 mL/min.


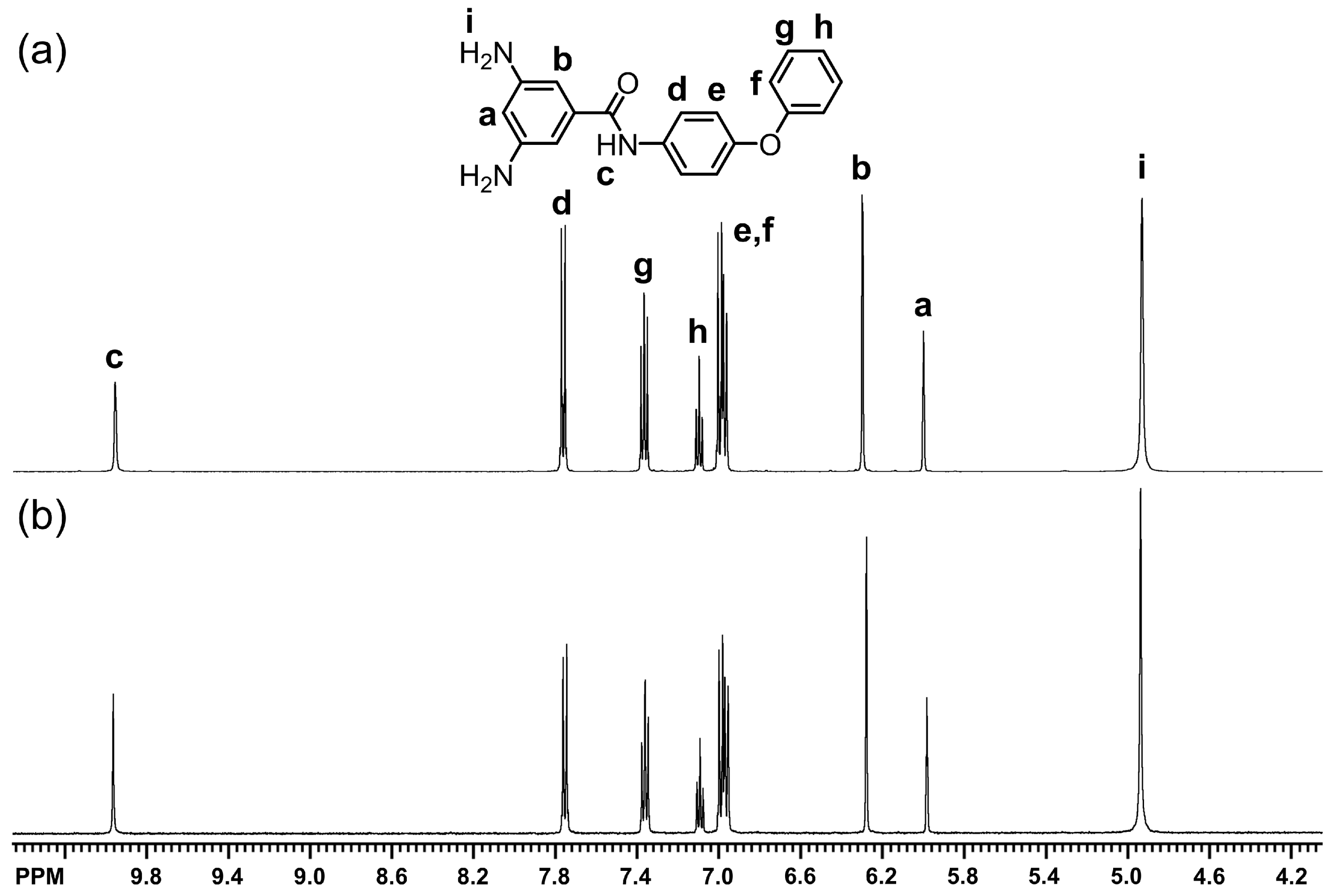


**Figure S4.** ^1^H NMR spectra of (a) ***m*-PDA** and (b) recovered ***m*-PDA** in DMSO-*d*_6_.
